# Supplementary material for: Risk of Recurrent Peptic Ulcer Disease in Patients Receiving Cumulative Defined Daily Dose of Nonsteroidal Anti-Inflammatory Drugs
Source: J Clin Med. 2019 Oct 18;8(10):1722. doi: 10.3390/jcm8101722 (PMC6833096; doi:10.3390/jcm8101722)
Supplement: Supplementary file 1 [file jcm-08-01722-s001.pdf]

**Table S1.** Summary of *Helicobacter pylori* treatment regimens.

| Type of Acid Suppression Used* | Combination of Antibiotics* |
| --- | --- |
| Proton pump inhibitor | Amoxicillin plus clarithromycin or metronidazole<br>Clarithromycin plus metronidazole<br>Tetracycline plus amoxicillin or clarithromycin or metronidazole<br>Levofloxacin plus amoxicillin or tetracycline |
| H2-blocker | Amoxicillin plus clarithromycin or metronidazole<br>Clarithromycin plus metronidazole<br>Tetracycline plus amoxicillin or clarithromycin or metronidazole<br>Levofloxacin plus amoxicillin or tetracycline |

*Patients prescribed with or without bismuth were all considered as *Helicobacter pylori* treatment regimens.
